# Melatonin and Its Analogs for Prevention of Post-cardiac Surgery Delirium: A Systematic Review and Meta-Analysis

**DOI:** 10.3389/fcvm.2022.888211

**Published:** 2022-05-18

**Authors:** Yunyang Han, Yu Tian, Jie Wu, Xiaoqin Zhu, Wei Wang, Zhenhua Zeng, Zaisheng Qin

**Affiliations:** ^1^Department of Anaesthesiology, Nanfang Hospital, Southern Medical University, Guangzhou, China; ^2^Department of Anesthesiology, Guangdong Women and Children Hospital, Guangzhou, China; ^3^Department of Critical Care Medicine, Nanfang Hospital, Southern Medical University, Guangzhou, China

**Keywords:** melatonin, cardiac, meta-analysis, ramelteon, systematic review, postoperative delirium

## Abstract

**Background:**

The effectiveness of melatonin and its analogs in preventing postoperative delirium (POD) following cardiac surgery is controversial. The purpose of this systematic review and meta-analysis was to confirm the benefits of melatonin and its analogs on delirium prevention in adults who underwent cardiac surgery.

**Methods:**

We systematically searched the PubMed, Cochrane Library, Web of Science, Embase, and EBSCOhost databases, the last search was performed in October 2021 and repeated before publication. The controlled studies were included if investigated the impact of melatonin and its analogs on POD in adults who underwent cardiac surgery. The primary outcome was the incidence of delirium. The Stata statistical software 17.0 was used to perform this study.

**Results:**

This meta-analysis included eight randomized controlled trials (RCTs) and two cohort studies with a total of 1,714 patients. The results showed that melatonin and ramelteon administration were associated with a significantly lower incidence of POD in adults who underwent cardiac surgery (odds ratio [OR], 0.46; 95% confidence interval [CI], 0.29–0.74; *P* = 0.001). The subgroup analyses confirmed that melatonin 3 mg (OR, 0.37; 95% CI, 0.18–0.76; *P* = 0.007) and 5 mg (OR, 0.34; 95% CI, 0.21–0.56; *P* < 0.001) significantly reduced the incidence of POD.

**Conclusion:**

Melatonin at dosages of 5 and 3 mg considerably decreased the risk of delirium in adults who underwent cardiac surgery, according to our results. Cautious interpretation of our results is important owing to the modest number of studies included in this meta-analysis and the heterogeneity among them.

**Systematic Review Registration:**

PROSPERO registration number: CRD42021246984.

## Introduction

Delirium, a neuropsychiatric disturbance characterized by a global impairment in cognitive capacity, including awareness and attention, deficient psychomotor activity, and a disrupted sleep-wake cycle (1), is associated with high mortality (2), cognitive decline (3), destruction of autonomy (4), an elevated hospitalization length and cost burden (5). Postoperative delirium (POD) is prevalent following cardiac surgery (6); the occurrence of post-cardiac surgery delirium ranges from 13 to 52% (7), and age, cognitive decline, weakness, complicated comorbidities, emergency operation, pain, and psychoactive medication administration are pivotal precipitating aspects (8). A recent study reported that a low left ventricular ejection fraction, atrial fibrillation, and cardiopulmonary bypass may predispose patients to post-cardiac surgery delirium (9).

The prevention of POD may play a significant role in patient recovery following cardiac surgery; although the pathophysiological pathways that cause delirium are yet unknown, The most prominent hypotheses regarding its neuropathogenesis include neuronal aging and neuroinflammation (10).

Melatonin (N-acetyl-5-methoxytryptamine), a pineal gland hormone, alleviates cognitive impairment (11, 12), protects against neurodegenerative diseases (13), and reduces neuroinflammation (14). When used for a cardioprotective medication, decreases heart dysfunction and improves the left ventricular ejection fraction in humans (15). Melatonin is commonly used in patients with sleep disorders and jetlag and has a good safety profile, even at large doses (16). Furthermore, Several melatonin analogs (tasimelteon, agomelatine, and ramelteon) are currently beneficial in the treatment of sleeplessness, depression, and circadian cycle disorders (17).

However, whether melatonin and its analogs prevent delirium in adults who underwent cardiac surgery remains unclear. Our previous meta-analysis indicated that melatonin and ramelteon (a melatonin analog) considerably decreased the occurrence of POD in adults; however, no statistically significant differences were observed in adults who underwent cardiac surgery (18). Several recent randomized controlled trials (RCTs) highlighted the advantage of melatonin for preventing post-cardiac surgery delirium (19–22). Herein, an up-to-date meta-analysis was conducted to see if melatonin and its analogs could reduce the risk of POD in cardiac surgery patients.

## Methods

This systematic review and meta-analysis of previously published research did not require ethical approval or patient permission. The PRISMA guidelines were followed for conducting and reporting this systematic review and meta-analysis (23). This study was registered on PROSPERO (CRD42021246984).

### Literature Search

We searched the PubMed, Cochrane Library, Web of Science, Embase, and EBSCOhost databases for studies investigating the impact of melatonin and its analogs on delirium prevention after cardiac surgery. We used combinations of the following search terms: (“melatonin receptor agonist OR N-acetyl-5-methoxytryptamine OR melatonin OR ramelteon OR tasimelteon OR agomelatine OR Melaxen OR melatonergics”) AND (“cardiac surgery OR cardiothoracic surgery OR coronary artery bypass OR surgical coronary revascularization OR valve surgery OR valve replacement”) AND (“delirium OR confusion OR confusional syndrome OR postoperative delirium OR cognitive disorder”). The last search was performed in October 2021 and repeated before publication (Supplementary Table 1).

### Study Selection

Based on an examination of the titles and abstracts, two reviewers (YYH and YT) separately selected possibly relevant articles. Differences of opinion were settled by consensus or discussion with a leading reviewer (ZSQ). We included controlled studies that investigated the impact of melatonin and its analogs on POD in adults who underwent cardiac surgery. Given the modest number of RCTs that have examined this topic, no predetermined limits on research design or quality were utilized to select studies. A previous study showed that well-designed observational studies did not exaggerate the degree of the treatment effects vs. RCTs that investigated the same topic (24). As a result, this study included RCTs, cohort studies, and case-control studies.

### Inclusion and Exclusion Criteria

The inclusion criteria were as follows:

(a) Study type: case-control studies, cohort studies, and RCTs;
(b) Publication language: Only English;
(c) Patients: Adults who underwent cardiac procedures;
(d) Intervention: Administration of melatonin and its analogs;
(e) Control: Placebos including lactose powder, sedatives including oxazepam, or a blank control;
(f) Outcomes: The primary outcome was the incidence of delirium, while the secondary outcomes were mechanical ventilation time, length of intensive care unit (ICU) stay, and length of hospitalization.

We excluded case reports, case reviews, conference reports, systematic reviews, and meta-analyses. Documentation management software (NoteExpress, version 3.4) was used to screen records and document decisions.

### Data Extraction and Quality Assessment

First author, publication year, number of patients, demographic characteristics, control group, type and dose of melatonin and its analogs, follow-up duration, diagnostic criteria of delirium, study design, incidence of delirium, mechanical ventilation time, length of ICU stay, and length of hospitalization were extracted from every study included in this meta-analysis. All of the retrieved data was collected in an Excel file. The risk of bias within RCTs was assessed using the revised Cochrane Collaboration Risk of Bias Tool (version 2.0) (25). To measure the quality of non-RCTs, the Newcastle-Ottawa Scale (NOS) was utilized (26). Two reviewers (QZS and YT) independently assessed each study, a third reviewers (HYY) was engaged in situations of disagreement.

### Statistical Analysis

The Stata statistical software (version 17.0; StataCorp, College Station, TX, USA) was used to perform this study. The restricted maximum likelihood random effects model was used to calculate pooled estimates and 95% confidence intervals (CIs). Dichotomous outcomes are expressed as odds ratios (ORs) with 95% CIs, while continuous outcomes were pooled using standard mean differences (SMD; Hedges' g) with 95% CIs. The median and interquartile range were converted to mean and standard deviation based on the quantile estimation method described by McGrath et al. (27). Tests for homogeneity based on Cochran's Q and *I*^2^ statistics were used; studies with an *I*^2^ > 50% were defined as those with moderate heterogeneity and those with an *I*^2^ > 75% were defined as those with significant heterogeneity (28). To find the origins of heterogeneity, subgroup analyses were used. Potential publication bias was assessed by visual inspection of funnel plots. In cases in which asymmetry was detected in the funnel plot, we used trim-and-fill analysis to assess publication bias. Leave-one-out meta-analysis was used to investigate the effects of each study on the overall effect size estimate and identify relevant studies. Two-tailed *P* < 0.05 were considered statistically significant for all analyses.

## Results

### Study Selection

**Figure 1:**
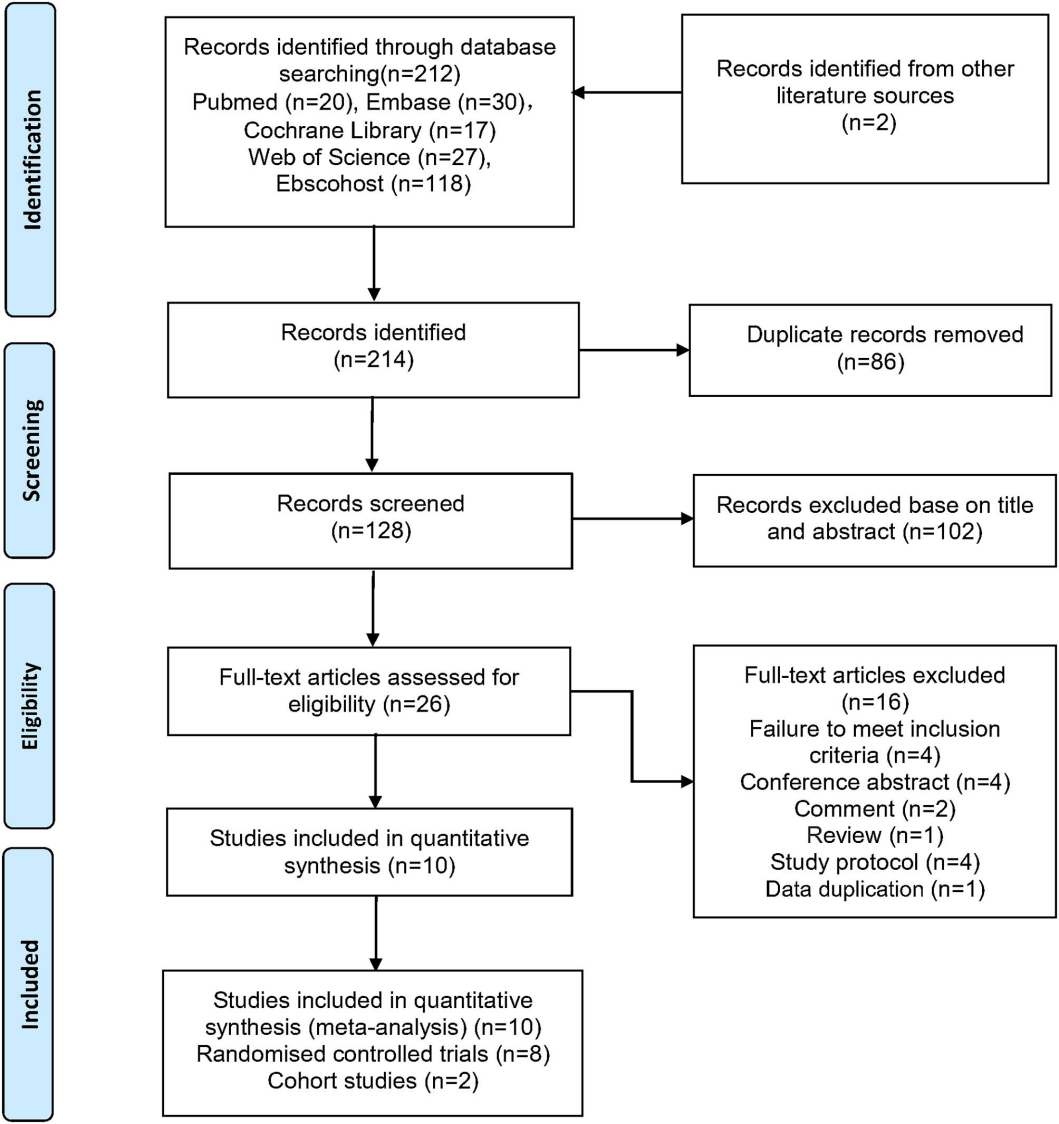
Flow diagram of literature search and study selection.

Figure 1 illustrates the process of study selection. Two independent researchers (YYH and YT) performed the literature search, data extraction, and general description of the included studies. We identified 214 studies published through October 30, 2021, including two records identified from other literature sources. We employed automated methods to eliminate 86 duplicate studies prior to screening. After analyzing the titles and abstracts, we determined that 26 full-text publications were possibly acceptable for inclusion, whereas 102 studies were excluded. Finally, eight RCTs (19–22, 29–32) and two observational studies (33, 34) were included based on the aforementioned eligibility criteria.

### Study Characteristics

Ten studies (19–22, 29–34) (1,714 patients) were published in English between 2015 and 2021. The sample size ranged from 50 to 500, and the participants were mainly elderly inpatients. The interventions used included ramelteon 8 mg in two studies (31, 34) and melatonin 3 mg or 5 mg in eight studies (19–22, 29, 30, 32, 33). Nine studies (19–21, 29–34) included 1,417 patients who underwent cardiothoracic surgery, such as coronary artery bypass graft surgery, aortic valve replacement, mitral valve replacement, and elective pulmonary thromboendarterectomy. A total of 297 patients underwent percutaneous transluminal coronary intervention (22). As diagnostic methods for POD, the Intensive Care Delirium Screening Checklist (ICDSC) (32, 34), Confusion Assessment Method for the Intensive Care Unit (CAM-ICU) (19–22, 30, 31, 33), and clinical observations (29) were employed. Table 1 presents the characteristics of the included studies.

**Table 1:** Characteristics of studies included in the meta-analysis.

**Referencs**	**Sample size**	**Male/ female**	**Age (years)**	**Drugs**	**Dose (mg)**	**Duration of preoperative melatonin administration**	**Follow-up (days)**	**Diagnose criteria**	**Delirium** **(%)**	**Surgery**	**Study design**
Artemiou et al. (33)	500	179/71 171/79	64.3 ± 10.1 65.2 ± 10.3	Prolonged-release tablets of melatonin, placebo	5	The evening before the operation	3 days	CAM-ICU	8.4 20.8	CABG, AVR, MVR, OPCAB, ASD, myxoma	Prospective cohort study
Dianatkhah et al. (29)	137	53/13 52/19	60.03 ± 10.21 61.70 ± 9.86	Melatonin, oxazepam	3	3 nights	Until the time of discharge	Clinical observation	6.1 12.7	CABG	RCT
Ford et al. (30)	210	79/26 85/25	69 (8.3) 67.7 (8)	Melatonin, placebo	3	2 nights	5 days	CAM-ICU	21.4 20.2	CABG	RCT
Hosseini Kasnavieh et al. (20)	140	57/13 61/9	64.03 64.5 (mean)	Melatonin, placebo	3	3 days	3 days	CAM-ICU	5.7 31.4	CABG	RCT
Jaiswal et al. (31)	117	29/29 29/30	58.1 ± 14.1 56.1 ± 15.8	Ramelteon, Placebo	8	The night before surgery	6 days	CAM-ICU	38 32	Elective pulmonary thromboendarterectomy	RCT
Javaherforoosh Zadeh et al. (19)	60	20/10 22/8	60.26 ± 9.50 62.9 ± 8.08	Prolonged-release tablets of melatonin, Placebo	3	The evening before the operation and the morning of surgery	1 day	CAM-ICU	10 46.6	CABG	RCT
Mahrose et al. (21)	110	42/13 41/14	67.0 ± 6.7 66.1 ± 6.3	Melatonin, none	5	10 PM, the night before surgery	6 days	CAM-ICU	10.9 27.3	CABG	RCT
Sharaf et al. (32)	50	12/13 14/11	66.56 (4.79) 67.88 (4.13)	Melatonin, placebo	3	Once at night and once 30 min preoperatively	3 days	ICDSC	8 28	CABG	RCT
Shi (22)	297	93/55 89/60	71.5 ± 6.7 71.6 ± 6.6	Melatonin, placebo	3	Postoperatively	7 days	CAM-ICU	27 39.6	PCI	RCT
Tamura et al. (34)	93	8/17 13/54	59.4 ± 12.3 68.0 ± 10.1	Ramelteon, none	8	Postoperatively	Until the time of discharge	ICDSC	26.9 29.9	CABG	Retrospective cohort study

*ASD, atrial septal defect; AVR, aortic valve replacement; CABG, coronary artery bypass graft surgery; CAM-ICU, Confusion Assessment Method for the Intensive Care Unit; ICDSC, Intensive Care Delirium Screening Checklist; MVR, mitral valve replacement; OPCAB, off-pump coronary artery bypass; PCI, percutaneous transluminal coronary intervention; RCT, randomized controlled trial*.

### Quality Assessment

Of the eight RCTs (19–22, 29–32), six were classified as low-risk (19, 20, 22, 30–32), and one was classified as being associated with “some concern” because the randomization process could not be blinded (21). The other study was also classified as being associated with “some concern” owing to deviations from intended interventions and outcome measurements (29) (Supplementary Table 2). The NOS scores in the two non-randomized observational studies were 8 (33) and 9 (34), respectively (Supplementary Table 3).

### Primary Outcome

**Figure 2:**
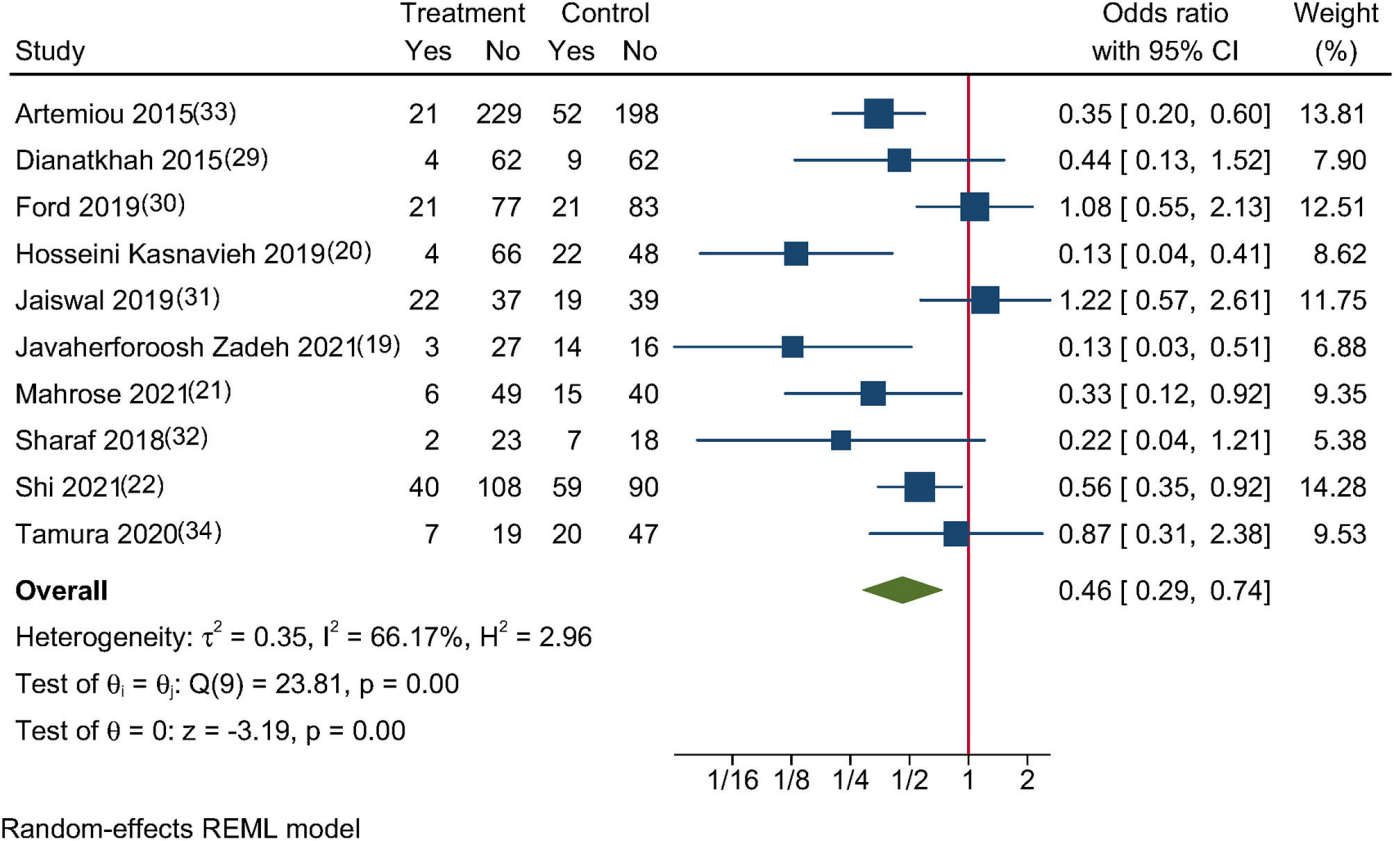
Forest plot for POD incidence in adults who underwent cardiac surgery.

This meta-analysis analyzed eight RCTs (19–22, 29–32) and two cohort studies (33, 34), which reported the incidence of POD. Melatonin and ramelteon use were linked to a considerably decreased incidence of POD in adults after cardiac surgery (OR, 0.46; 95% CI, 0.29–0.74; *P* = 0.001; Figure 2), with moderate heterogeneity across studies that reported these findings (*I*^2^ = 66.17%; *P* = 0.005).

#### Subgroup Analyses and Leave-One-Out Meta-Analysis

**Figure 3:**
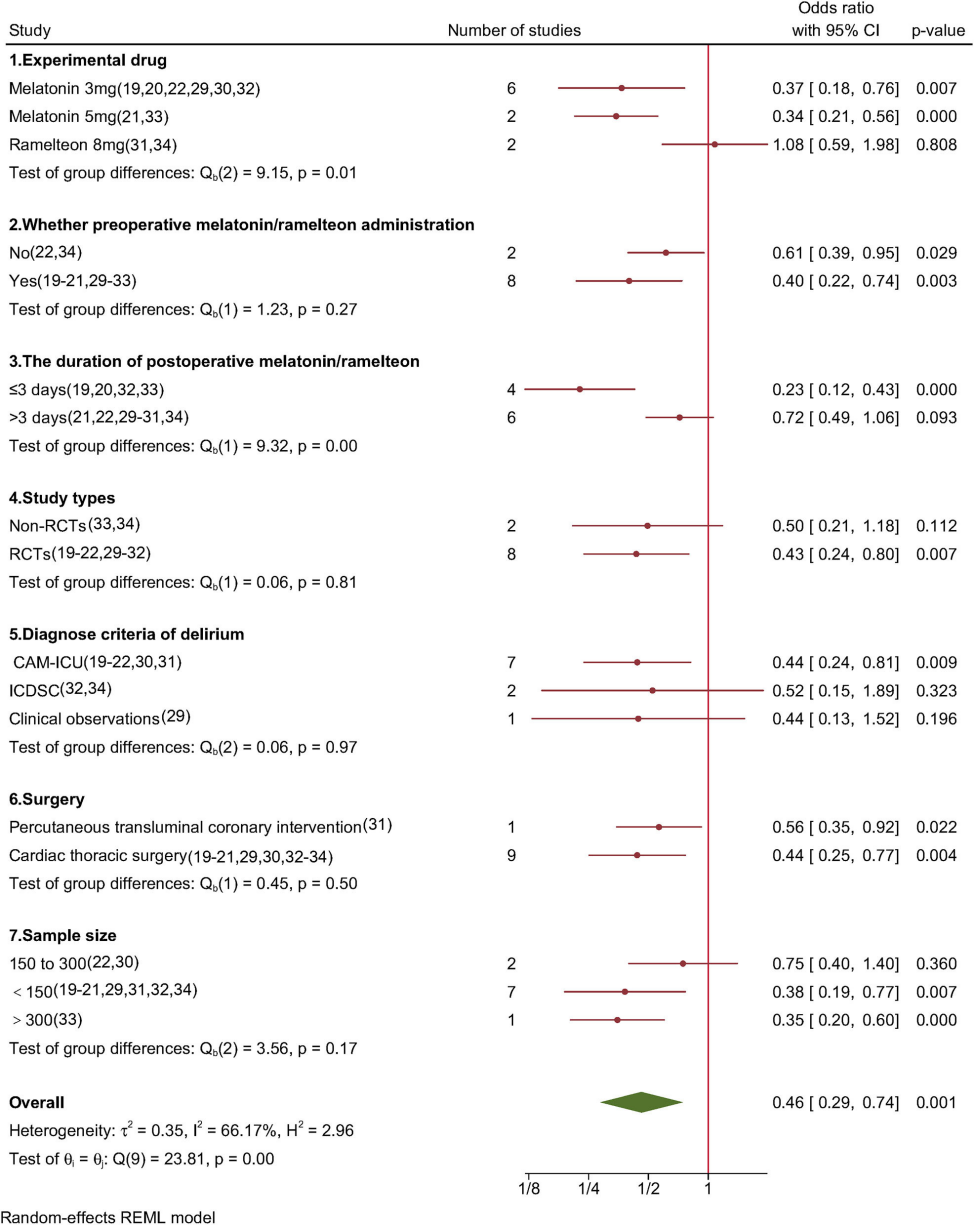
Forest plot of subgroup analysis for POD incidence in adults who underwent cardiac surgery.

Figure 3 shows the outcomes of the subgroup analyses, which showed that the incidence of POD was reduced in both melatonin groups (3 mg group [OR, 0.37; 95% CI, 0.18–0.76; *P* = 0.007] and the 5 mg group [OR, 0.34; 95% CI, 0.21–0.56; *P* < 0.001]); however, no such effect was observed in the ramelteon 8 mg group (OR, 1.08; 95% CI, 0.59–1.98; *P* = 0.808). The subgroup analysis showed that preoperative melatonin/ramelteon administration (OR, 0.40; 95% CI, 0.22–0.74; *P* = 0.003) was more beneficial for POD prevention than postoperative administration (OR, 0.61; 95% CI, 0.39–0.95; *P* = 0.029). Significant differences in the incidence of POD were observed in the short-term (≤3 days) group (OR, 0.23; 95% CI, 0.12–0.43; *P* < 0.001) but not in the long-term (>3 days) group (OR, 0.72; 95% CI, 0.49–1.06; *P* = 0.093). Significant subgroup heterogeneity was observed in the experimental drug (*P* = 0.01) and postoperative duration (*P* < 0.01) of melatonin/ramelteon administration.

Further subgroup analysis showed that the RCT group (OR, 0.43; 95% CI, 0.24–0.80; *P* = 0.007) had a lower incidence of POD, but the non-RCT group did not show any significant differences (OR, 0.50; 95% CI, 0.21–1.18; *P* = 0.112). Additionally, a subgroup analysis based on study type, controlled drugs, diagnostic criteria for delirium, surgery, and sample size showed no significant heterogeneity. The overall results were steady, and no study was recognized as an outlier based on the leave-one-out method's findings (Supplementary Figure 1).

#### Publication Bias

Some degree of asymmetry in the funnel plot suggested publication bias (Supplementary Figure 2). To estimate the missing studies, we employed the trim-and-fill procedure and recalculated the total pooled effect estimates. The plot revealed that the two imputed studies fell in the gray and dark gray regions, corresponding to *P* > 0.05. The addition of imputed studies to the meta-analysis increased the overall OR from 0.46 to 0.58 (Supplementary Figure 3). These results support the conclusion that the small study effect is most likely attributable to publication bias.

#### Secondary Outcomes

**Figure 4:**
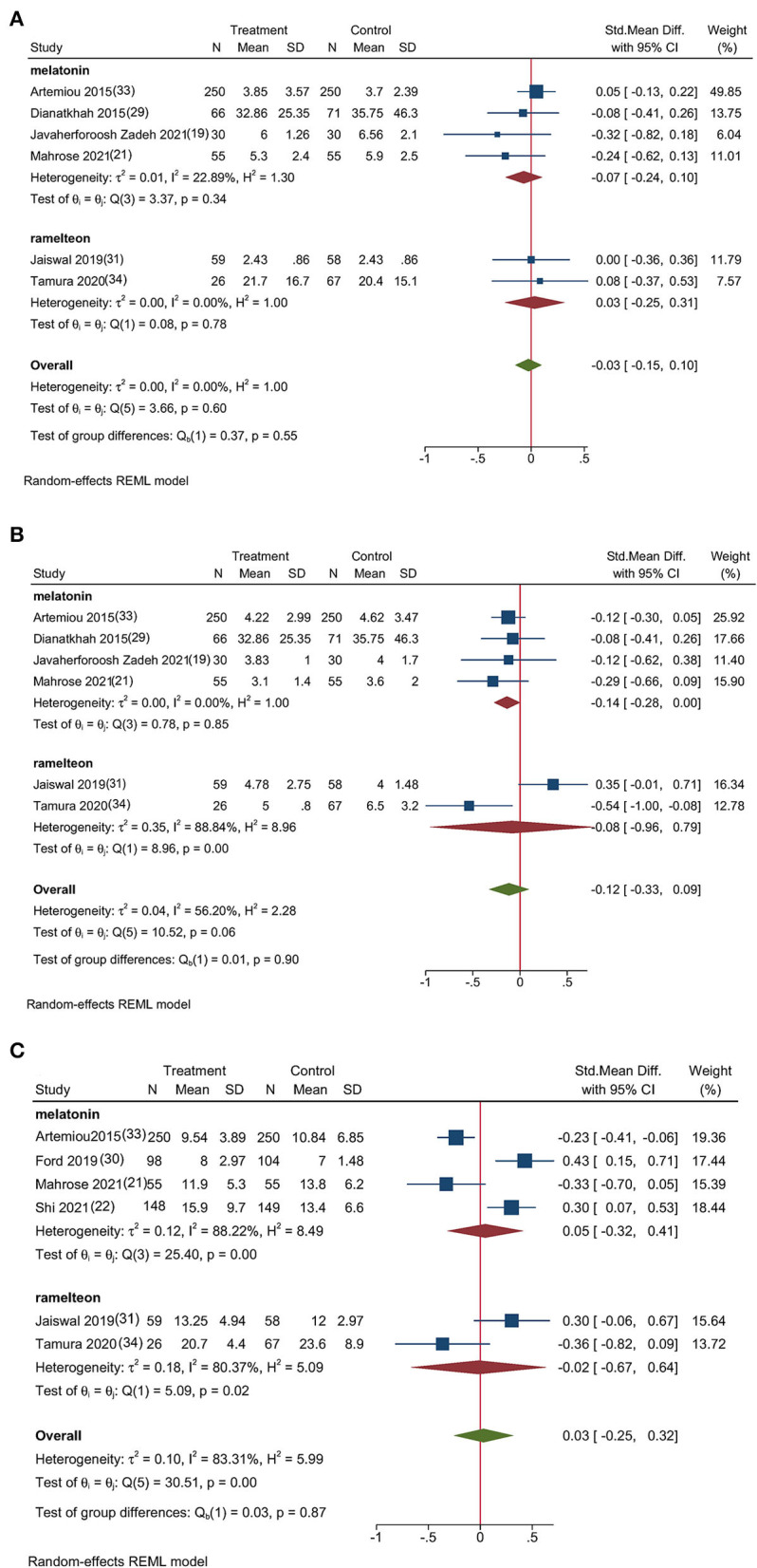
Forest plot of the secondary outcomes; **(A)** Forest plot of the mechanical ventilation time, **(B)** Forest plot of the length of ICU stay, and **(C)** Forest plot of the length of hospitalization.

Figure 4 shows the secondary outcomes of this study. Six studies (19, 21, 29, 31, 33, 34) (1,017 participants) reported the duration of postoperative mechanical ventilation, and the meta-analysis showed that mechanical ventilation time was not significantly shortened following melatonin or ramelteon administration (SMD, −0.03; 95% CI, −0.15 to 0.10; *P* = 0.60; Figure 4A). A meta-analysis of seven studies (19, 21, 29, 31, 33, 34) (1,017 patients) reported that melatonin or ramelteon administration did not significantly shorten the length of ICU stay (SMD, −0.12; 95% CI, −0.33 to 0.09; *P* = 0.06; Figure 4B). A pooled analysis of six studies (21, 22, 30, 31, 33, 34) (1,319 participants) showed no significant shortening of the length of hospitalization following melatonin or ramelteon administration (SMD, 0.03; 95% CI, −0.25 to 0.32; *P* < 0.001, Figure 4C). A subgroup analysis following melatonin and ramelteon administration revealed no significant intergroup differences in any of the secondary outcomes.

## Discussion

The present meta-analysis showed that the administration of melatonin 5 or 3 mg could decrease the risk of POD in adults who underwent cardiac surgery. However, the administration of melatonin or ramelteon did not shorten the duration of postoperative mechanical ventilation, length of ICU stay, or length of hospitalization. Moreover, owing to the small number of included studies and high heterogeneity among them, the results should be interpreted with caution.

A subgroup analysis of our previous meta-analysis of four studies (29, 30, 32, 33) (889 patients) indicated that melatonin administration did not significantly minimize the incidence of delirium in adults who underwent cardiac surgery (18). However, the results of cardiac surgery subgroup analyses from the small sample size and restricted number of included studies may have low power to detect such a difference. The present meta-analysis included six of the most recently published studies (19–22, 31, 34) based on previous research, and the results indicated that melatonin might efficiently prevent POD in adults who underwent cardiac surgery; however, this finding is not consistent with those reported by previous studies. However, the present meta-analysis' results were based on a bigger sample size than the prior study.

The melatonin dose varied from 0.5 to 5 mg across studies for delirium prevention (35). A recent expert consensus recommended that the administration of prolonged-release melatonin 2 mg or an immediate-release 3 or 5 mg dose before bedtime may prevent the incidence of delirium in at-risk populations (36). However, the dose of melatonin that prevents POD has not been systematically determined in adults who underwent cardiac surgery. This meta-analysis showed that melatonin 5 and 3 mg could effectively reduce the incidence of POD, while extended-release melatonin 5 and 3 mg reduced delirium risk by 12.4% (33) and 36.6% (19), respectively. None of the included studies reported serious adverse events associated with the use of melatonin, such as respiratory suppression, hemodynamic fluctuations. A network meta-analysis of six RCTs reported that the administration of melatonin 0.5 mg was associated with a better preventive effect than administration of the same dose of melatonin in adult inpatients (37); however, the effect of low-dose melatonin on the development of delirium in patients who underwent cardiac surgery remains unclear. Therefore, further RCTs are warranted to establish the optimal melatonin dose for POD prevention.

Dexmedetomidine is also a hot topic drug in recent clinical studies on POD prevention. Melatonin and dexmedetomidine may differ in the prevention of POD as follows: (a) Dexmedetomidine is an α2-adrenoceptor agonist with sedative, anxiolytic, and sympatholytic effects. Perioperative dexmedetomidine administration was associated with an elevated risk of bradycardia and hypotension (38) resulted from sympatholytic properties. Melatonin is commonly used in patients with sleep disorders and jetlag and has a good safety profile, even at large doses (≥10 mg) (16). (b) Dexmedetomidine needs to be administered intravenously under the supervision of medical personnel, and melatonin needs to be administered orally autonomously before or after surgery. (c) In contrast to dexmedetomidine, which requires a doctor's prescription, melatonin is sold as an over-the-counter drug, such as in the United States and China. (d) Multiple randomized controlled trials (RCTs) have demonstrated that the administration of dexmedetomidine is associated with a lower incidence of POD following non-cardiac surgery (39–41), however, dexmedetomidine was not associated with decreased incidence of POD following cardiac surgery (42, 43). The different pharmacological mechanisms of dexmedetomidine and melatonin may lead to different effects of dexmedetomidine on POD.

Ramelteon, the only melatonin analog included in this meta-analysis, has a 6-fold higher affinity for melatonin 1 receptors and a 3-fold higher affinity for melatonin 2 receptors (44); ramelteon's higher affinity for melatonin receptors may contribute to its favorable benefits in delirium prevention, and previous RCTs reported that ramelteon can reduce POD after non-cardiac surgery in elderly patients (45, 46). However, in this meta-analysis, results from an RCT and an observational study showed no evidence of a preventive effect of ramelteon 8 mg on POD in adults who underwent cardiac surgery.

Ramelteon has an overall absorption rate of 84%, although its total bioavailability is just 1.8% (47); the bioavailability of oral melatonin was ~15% (48). The fasting state is an important cause of inter-individual variability in drug bioavailability (49) and a possible reason for the difference in the effectiveness of melatonin and ramelteon for POD prevention. Only two studies (31, 34) on ramelteon were included in this meta-analysis; thus, it remains unclear whether it can prevent delirium in patients who undergo cardiac surgery.

The exact mechanism of post-cardiac surgery delirium is uncertain and may be of multifactorial origin (50). But Previous studies have suggested that old age (51) and inflammation (52) are independent risk factors for POD. Neuronal aging and neuroinflammation may be the underlying mechanisms (10). Neuroinflammation disrupts the brain network of executive function after cardiac surgery (53). Aging neurons inevitably affect the brain's normal functional reserves (54). Melatonin may play an important role in the suppression of neuroinflammation (55) and the treatment of age-related neurodegenerative diseases (13).

Although different doses of melatonin show the same preventive effect on delirium, preoperative melatonin administration appears to produce a more significant effect, possible due to the following factors: (a) Sleep disorder is associated with a high risk for delirium (56), and patients with various cardiac diseases invariably experience preoperative sleep disorders secondary to anxiety, depression, breathing disorders, or other factors (57); (b) The secretion of melatonin is delayed during anesthesia and surgery (58); (c) Postoperatively given melatonin absorption kinetics are greatly slowed, and peak plasma levels are reduced (59); and (d) Anesthesia and surgery may selectively cause functional reductions in the prefrontal cortex excitatory synaptic transmission and induce delirium (60). It is reasonable to initiate melatonin supplementation the night before cardiac surgery if conditions permit. A recent study indicated that most cases of delirium occur between days 1 and 3 after cardiac surgery (61); which may explain the lack of a significant difference observed in the long-term group. The mean duration of POD was 3 days after cardiac surgery (62). Therefore, it may be appropriate to administer melatonin preoperatively and continue its administration until postoperative day 6.

In patients undergoing cardiac surgery, the incidence of delirium is strongly associated with the duration of mechanical ventilation, ICU stay, and hospitalization. POD can prolong the mechanical ventilation time, length of ICU stay, length of hospitalization after cardiac surgery (50, 63, 64), and duration of mechanical ventilation, length of ICU stay, and length of hospitalization serve as precipitating factors for POD (65, 66). However, our meta-analysis did not show a significant difference in the aforementioned clinical outcomes based on the premise that melatonin can significantly reduce the incidence of POD. The limited number of patients included in the meta-analysis may have contributed to this observation; however, it is more likely that differences in delirium severity, duration, and subtype (including hypoactive, hyperactive, and mixed) may have resulted in the differences in clinical outcomes. A high delirium severity score (67), prolonged delirium (68), and hypoactive delirium (63, 69) were most significantly associated with the duration of mechanical ventilation, length of ICU stay, and length of hospitalization.

The potential limitations of this study are as follows: (a) Owing to the scarcity of studies that have investigated this topic, RCTs and observational studies were included to enhance the robustness of this meta-analysis. Although the subgroup analysis revealed that the non-RCT group did not overestimate the preventive effect of melatonin on POD, and the result was not changed in leave-one-out meta-analysis excluding each observational study individually, observational studies are prone to selection bias secondary to a lack of randomization. (b) Because of the inclusion of studies with small sample sizes, and the asymmetry of the funnel plot and the trim-and-fill approach imply that the current meta-analysis may be prone to small-study effect bias. To definitively confirm the effects of melatonin and ramelteon on POD, more large-scale investigations are required. (c) The studies included in the meta-analysis showed moderate heterogeneity. The subgroup analyses indicated that “the type and dose of melatonin/ramelteon” and “whether preoperative melatonin/ramelteon was used” may have contributed to the heterogeneity. These variables may have an impact on the precision and consistency of the results. (d) No reliable laboratory parameters/biomarkers are available for the diagnosis of delirium, and the delirium screening tools used by the included studies differed. Both the CAM-ICU and the ICDSC are accurate assessment tools for identifying delirium in critically ill patients, the CAM-ICU is superior at ruling out patients without delirium in the ICU and detecting delirium in patients receiving mechanical ventilation (70), this difference may affect the incidence of POD since most patients undergoing open heart surgery require mechanical ventilation after surgery in the ICU. Additional evidence from well-designed large-scale RCTs is required to validate these findings.

## Conclusion

This study revealed that melatonin 5 and 3 mg considerably decreased the risk of delirium in adults who underwent cardiac surgery. However, ramelteon 8 mg did not exhibit this effect. Moreover, the duration of postoperative mechanical ventilation, length of ICU stay, and length of hospitalization were not significantly affected by melatonin or ramelteon administration. A cautious interpretation of these results is necessary considering the modest number of studies included in this meta-analysis and the heterogeneity among them.

## Data Availability Statement

The original contributions presented in the study are included in the article/Supplementary Material, further inquiries can be directed to the corresponding authors.

## Author Contributions

YH conceived of the study, executed a search strategy, collected and analyzed the data, assessed the articles, interpreted the findings, and drafted the manuscript. YT executed a search strategy, collected and analyzed the data, assessed the articles, interpreted the findings, and drafted the manuscript. JW analyzed the data, assessed the articles, interpreted the findings, and drafted the manuscript. XZ and WW interpreted the findings and helped to prepare the manuscript. ZZ assisted in the execution of the study, data analysis, interpretation, and critical revision of the manuscript. ZQ assisted in the study design, data search and analysis, and critical revision of the manuscript. All authors contributed to the article and approved the submitted version.

## Funding

This work was supported by the National Natural Science Foundation of China (grant/award numbers: 81973305, 81571358, and 82172175).

## Conflict of Interest

The authors declare that the research was conducted in the absence of any commercial or financial relationships that could be construed as a potential conflict of interest.

## Publisher's Note

All claims expressed in this article are solely those of the authors and do not necessarily represent those of their affiliated organizations, or those of the publisher, the editors and the reviewers. Any product that may be evaluated in this article, or claim that may be made by its manufacturer, is not guaranteed or endorsed by the publisher.
