# Supplementary material for: Melatonin and Its Analogs for Prevention of Post-cardiac Surgery Delirium: A Systematic Review and Meta-Analysis
Source: Front Cardiovasc Med. 2022 May 18;9:888211. doi: 10.3389/fcvm.2022.888211 (PMC9157569; doi:10.3389/fcvm.2022.888211)
Supplement: Supplementary file 1 [file Data_Sheet_1.PDF]

Supplementary Table 1. Search Strategies for database.

PubMed Results:20

Search number	Search Details
#1	(("receptors, melatonin"[MeSH Terms] OR ("receptors"[All Fields] AND "melatonin"[All Fields]) OR "melatonin receptors"[All Fields] OR ("melatonin"[All Fields] AND "receptor"[All Fields]) OR "melatonin receptor"[All Fields]) AND ("agonist"[All Fields] OR "agonist s"[All Fields] OR "agonistic"[All Fields] OR "agonistically"[All Fields] OR "agonistics"[All Fields] OR "agonists"[MeSH Subheading] OR "agonists"[All Fields])) OR ("melatonin"[MeSH Terms] OR "melatonin"[All Fields] OR "n acetyl 5 methoxytryptamine"[All Fields]) OR ("melatonin"[MeSH Terms] OR "melatonin"[All Fields] OR "melatonin s"[All Fields] OR "melatonine"[All Fields] OR "melatonins"[All Fields]) OR ("ramelteon"[Supplementary Concept] OR "ramelteon"[All Fields]) OR ("tasimelteon"[Supplementary Concept] OR "tasimelteon"[All Fields]) OR ("s 20098"[Supplementary Concept] OR "s 20098"[All Fields] OR "agomelatine"[All Fields] OR "agomelatin"[All Fields] OR "agomelatine s"[All Fields]) OR "Melaxen"[All Fields] OR "Melatonergics"[All Fields]
#2	"thoracic surgery"[MeSH Terms] OR ("thoracic"[All Fields] AND "surgery"[All Fields]) OR "thoracic surgery"[All Fields] OR ("cardiac"[All Fields] AND "surgery"[All Fields]) OR "cardiac surgery"[All Fields] OR "cardiac surgical procedures"[MeSH Terms] OR ("cardiac"[All Fields] AND "surgical"[All Fields] AND "procedures"[All Fields]) OR "cardiac surgical procedures"[All Fields] OR ("cardiac"[All Fields] AND "surgery"[All Fields]) OR ("cardiothoracic"[All Fields] AND ("surgery"[MeSH Subheading] OR "surgery"[All Fields] OR "surgical procedures, operative"[MeSH Terms] OR ("surgical"[All Fields] AND "procedures"[All Fields] AND "operative"[All Fields]) OR "operative surgical procedures"[All Fields] OR "general surgery"[MeSH Terms] OR ("general"[All Fields] AND "surgery"[All Fields]) OR "general surgery"[All Fields] OR "surgery s"[All Fields] OR "surgerys"[All Fields] OR "surgeries"[All Fields])) OR ("coronary artery bypass"[MeSH Terms] OR ("coronary"[All Fields] AND "artery"[All Fields] AND "bypass"[All Fields]) OR "coronary artery bypass"[All Fields]) OR (("surgical procedures, operative"[MeSH Terms] OR ("surgical"[All Fields] AND "procedures"[All Fields] AND "operative"[All Fields]) OR "operative surgical procedures"[All Fields] OR "surgical"[All Fields] OR "surgically"[All Fields] OR "surgicals"[All Fields]) AND ("coronaries"[All Fields] OR "heart"[MeSH Terms] OR "heart"[All Fields] OR "coronary"[All Fields]) AND ("revascularisation"[All Fields] OR "revascularisations"[All Fields] OR "revascularise"[All Fields] OR "revascularised"[All Fields] OR "revascularising"[All Fields] OR "revascularization"[All Fields] OR "revascularizations"[All Fields] OR "revascularize"[All Fields] OR "revascularized"[All Fields] OR "revascularizes"[All Fields] OR "revascularizing"[All Fields])) OR (("valve"[All Fields] OR "valve s"[All Fields] OR "valved"[All Fields] OR "valves"[All Fields] OR "valving"[All Fields]) AND ("surgery"[MeSH Subheading] OR "surgery"[All Fields] OR "surgical procedures, operative"[MeSH Terms] OR ("surgical"[All Fields] AND "procedures"[All Fields] AND "operative"[All Fields]) OR "operative surgical procedures"[All Fields] OR "general surgery"[MeSH Terms] OR ("general"[All Fields] AND "surgery"[All Fields]) OR "general surgery"[All Fields] OR "surgery s"[All Fields] OR "surgerys"[All Fields] OR "surgeries"[All Fields])) OR (("valve"[All Fields] OR "valve s"[All Fields] OR "valved"[All Fields] OR "valves"[All Fields] OR "valving"[All Fields]) AND ("replace"[All Fields] OR "replaceable"[All Fields] OR "replaced"[All Fields] OR "replaces"[All Fields] OR "replacing"[All Fields] OR "replacment"[All Fields] OR "replantation"[MeSH Terms] OR "replantation"[All Fields] OR "replacement"[All Fields] OR "replacements"[All Fields]))

#3	"delirium"[MeSH Terms] OR "delirium"[All Fields] OR "deliriums"[All Fields] OR ("confusability"[All Fields] OR "confusable"[All Fields] OR "confuse"[All Fields] OR "confuses"[All Fields] OR "confusing"[All Fields] OR "confusion"[MeSH Terms] OR "confusion"[All Fields] OR "confused"[All Fields] OR "confusions"[All Fields]) OR ("confusional"[All Fields] AND ("syndrom"[All Fields] OR "syndromal"[All Fields] OR "syndromally"[All Fields] OR "syndrome"[MeSH Terms] OR "syndrome"[All Fields] OR "syndromes"[All Fields] OR "syndrome s"[All Fields] OR "syndromic"[All Fields] OR "syndroms"[All Fields])) OR (("postoperative period"[MeSH Terms] OR ("postoperative"[All Fields] AND "period"[All Fields]) OR "postoperative period"[All Fields] OR "postop"[All Fields] OR "postoperative"[All Fields] OR "postoperatively"[All Fields] OR "postoperatives"[All Fields]) AND ("delirium"[MeSH Terms] OR "delirium"[All Fields] OR "deliriums"[All Fields])) OR ("cognition disorders"[MeSH Terms] OR ("cognition"[All Fields] AND "disorders"[All Fields]) OR "cognition disorders"[All Fields] OR ("cognitive"[All Fields] AND "disorder"[All Fields]) OR "cognitive disorder"[All Fields])
#4	(#1) AND (#2)) AND (#3)

Cochrane Library Results:17

Search number	Search Details
1	(melatonin AND receptor AND agonist OR n-acetyl-5-methoxytryptamine OR melatonin OR ramelteon OR tasimelteon OR agomelatine OR melaxen OR melatonergics)
2	(cardiac AND surgery OR (cardiothoracic AND surgery) OR (coronary AND artery AND bypass) OR (surgical AND coronary AND revascularization) OR (valve AND surgery) OR (valve AND replacement)
3	(delirium OR confusion OR (confusional AND syndrome) OR (postoperative AND delirium) OR (valve AND surgerycognitive AND disorder)
4	1 AND 2 AND 3

Web of Science Results:27

Search number	Search Details
#1	ALL FIELDS: (melatonin receptor agonist) OR ALL FIELDS: (N-acetyl-5-methoxytryptamine) OR ALL FIELDS: (melatonin) OR ALL FIELDS: (ramelteon) OR ALL FIELDS: (tasimelteon) OR ALL FIELDS: (agomelatine) OR ALL FIELDS: (Melaxen) OR ALL FIELDS: (Melatonergics)<br>Indexes=SCI-EXPANDED, SSCI, A&HCI, CPCI-S, CPCI-SSH, BKCI-S, BKCI-SSH, ESCI, CCR-EXPANDED, IC Timespan=All years
#2	ALL FIELDS: (cardiac surgery) OR ALL FIELDS: (cardiothoracic surgery) OR ALL FIELDS: (coronary artery bypass) OR ALL FIELDS: (surgical coronary revascularization) OR ALL FIELDS: (valve surgery) OR ALL FIELDS: (valve replacement)<br>Indexes=SCI-EXPANDED, SSCI, A&HCI, CPCI-S, CPCI-SSH, BKCI-S, BKCI-SSH, ESCI, CCR-EXPANDED, IC Timespan=All years
#3	ALL FIELDS: (delirium) OR ALL FIELDS: (confusion) OR ALL FIELDS: (confusional syndrome) OR ALL FIELDS: (postoperative delirium) OR ALL FIELDS: (cognitive disorder)<br>Indexes=SCI-EXPANDED, SSCI, A&HCI, CPCI-S, CPCI-SSH, BKCI-S, BKCI-SSH, ESCI, CCR-EXPANDED, IC Timespan=All years
#4	#3 AND #2 AND #1<br>Indexes=SCI-EXPANDED, SSCI, A&HCI, CPCI-S, CPCI-SSH, BKCI-S, BKCI-SSH, ESCI, CCR-EXPANDED, IC Timespan=All years

Embase Results:30

Search number	Search Details
1	(melatonin AND receptor AND agonist OR n-acetyl-5-methoxytryptamine OR melatonin OR ramelteon OR tasimelteon OR agomelatine OR melaxen OR melatonergics)
2	(cardiac AND surgery OR (cardiothoracic AND surgery) OR (coronary AND artery AND bypass) OR (surgical AND coronary AND revascularization) OR (valve AND surgery) OR (valve AND replacement)
3	(delirium OR confusion OR (confusional AND syndrome) OR (postoperative AND delirium) OR (valve AND surgerycognitive AND disorder)
4	1 AND 2 AND 3<br>Mapped terms n/a

Ebscohost Results:118

Search number	Search Details
S1	(TX+delirium+OR+TX+confusion+OR+TX+confusional+syndrome+OR+TX+postoperative+delirium+OR+TX+cognitive+disorder)
S2	(TX+melatonin+receptor+agonist)+OR+(TX+N%E2%80%90acetyl%E2%80%905%E2%80%90methoxytryptamine)+OR+(TX+melatonin)+OR+(TX+ramelteon)+OR+(TX+tasimelteon)+OR+(TX+agomelatine)+OR+(TX+Melaxen)+OR+(TX+Melatonergics)
S3	(TX+delirium)+OR+(TX+confusion)+OR+(TX+confusional+syndrome)+OR+(TX+postoperative+delirium)+OR+(TX+cognitive+disorder)
S4	S1 AND S2 AND S3 (lang=zh-cn&type=0&searchMode=Standard&site=ehost-live)

Supplementary Table 2. Risk of bias assessment for RCTs

Author, Year	Randomization process	Deviations from intended interventions	Missing outcome data	Measurement of the outcome	Selection of the reported result	Overall
Dianatkhah 2015(29)	+	?	+	?	+	?
Ford 2019(30)	+	+	+	+	+	+
Hosseini Kasnavieh 2019(20)	+	+	+	+	+	+
Jaiswal 2019(31)	+	+	+	+	+	+
Javaherforoosh Zadeh 2021(19)	+	+	+	+	+	+
Mahrose 2021(21)	?	+	+	+	+	?
Sharaf 2018(32)	+	+	?	+	+	+
Shi 2021(22)	+	+	+	+	+	+

+ Low risk

? Some concerns

? High risk

Supplementary Table 3. Risk of bias assessment for non-RCTs

study	Study type	Selection	Comparability	Outcome or Exposure	Total score
Artemiou 2015(33)	prospective cohort study	****	*	***	8
Tamura 2020(34)	retrospective cohort study	****	**	***	9

Supplementary Figure 1. Forest plot for leave-one-out meta-analysis.

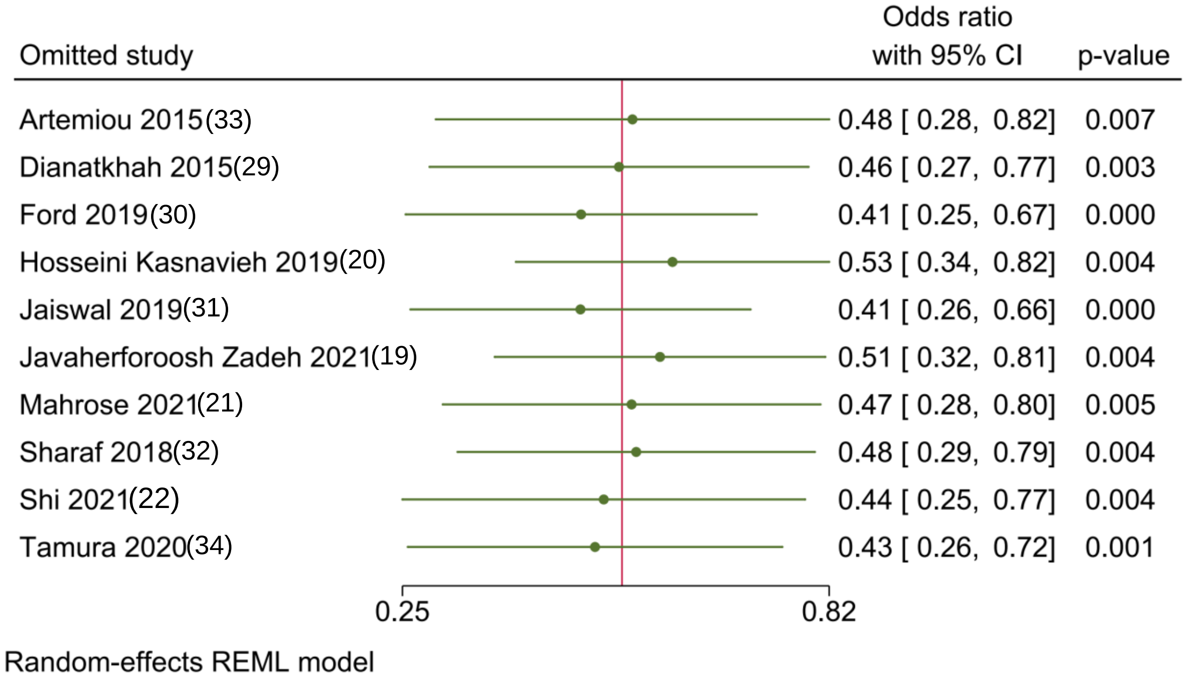

Supplementary Figure 2. Funnel plot for postoperative delirium incidence.

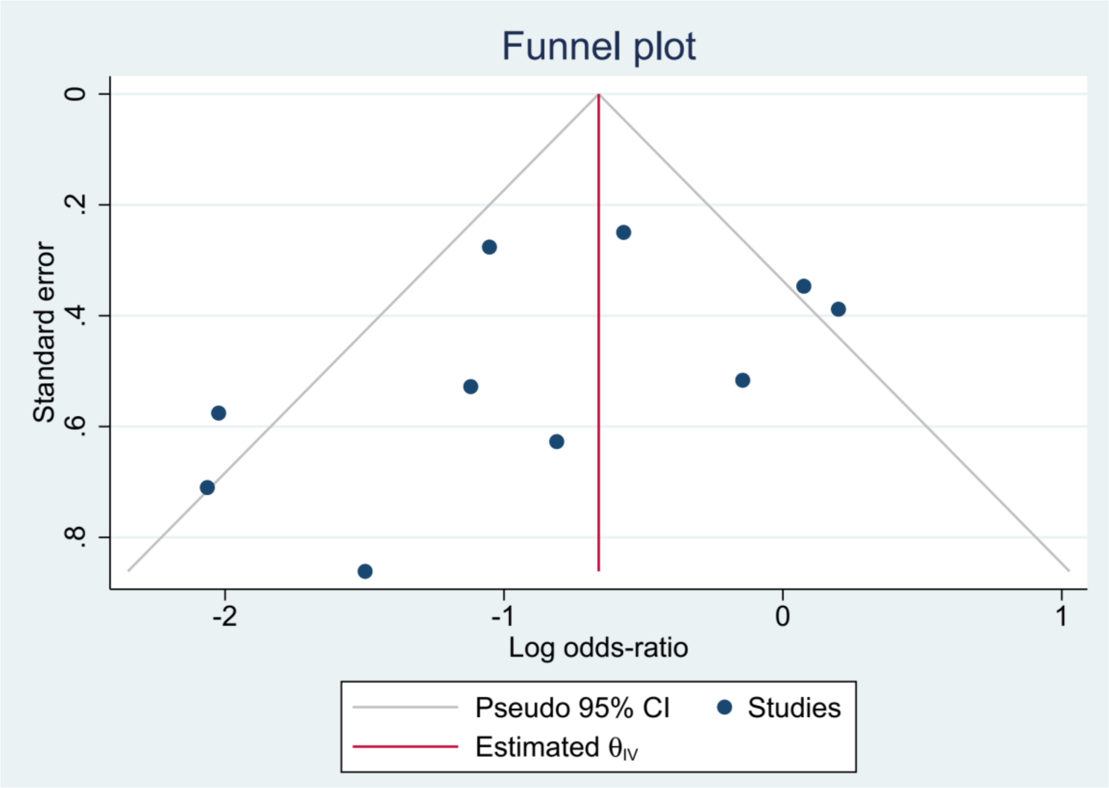

Supplementary Figure 3. Trim-and fill meta-analysis.

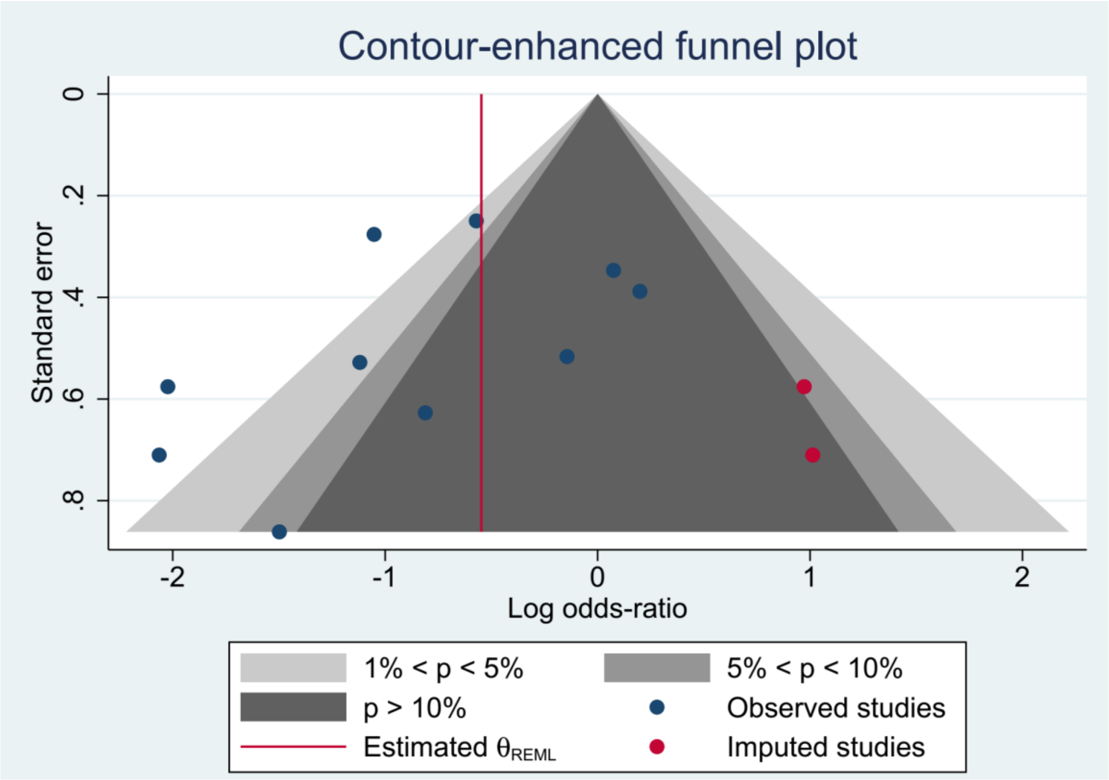

```
. meta trimfill, estimator(linear) eform

  Effect-size label:  Log Odds-Ratio
        Effect size:  _meta_es
          Std. Err.:  _meta_se

Nonparametric trim-and-fill analysis of publication bias
Linear estimator, imputing on the right

Iteration                            Number of studies =     12
  Model: Random-effects                       observed =     10
 Method: REML                                 imputed =      2

Pooling
  Model: Random-effects
 Method: REML

--------------------------------------------------------------
             Studies |     Odds Ratio    [95% Conf. Interval]
---------------------+----------------------------------------
            Observed |          0.461        0.287        0.742
  Observed + Imputed |          0.579        0.340        0.983
--------------------------------------------------------------
```
